# Bacterial Sepsis among Children with Congenital Heart Disease in Tikur Anbessa Specialized Hospital, Addis Ababa, Ethiopia

**DOI:** 10.4314/ejhs.v32i3.7

**Published:** 2022-05

**Authors:** Edomgenet Tesfaye, Henok Tadele

**Affiliations:** 1 Department of Pediatrics and Child Health, School of Medicine, College of Health Sciences, Addis Ababa University

**Keywords:** Congenital heart disease, bacterial sepsis, Down syndrome, Ethiopia

## Abstract

**Background:**

Bacterial Sepsis is a serious medical problem affecting children with Congenital Heart Disease (CHD). The pattern and factors predicting outcome of bacterial sepsis have not been studied in Africa. The study aimed to describe the pattern and outcome of bacterial sepsis among children with CHD in Tikur Anbessa Specialized Hospital (TASH).

**Methods:**

A cross-sectional study was carried out among children with CHD and sepsis at TASH between May 2017 and July 2020. Structured questionnaires were used for data collection. Statistical significance was set at P value < 0.05, and multivariable logistic regression was used to determine predictors.

**Results:**

This study included 384 CHD children with sepsis. Proportion of culture proven bacterial sepsis was 17.1 % (66) (95% CI: 13.6–21.3). Coagulase negative staphylococcus aureus 7% (27), Staphylococcus aureus 4.4% (17) and Actinobacteria 1.8% (7) were the common isolated bacteriological agents. Death was documented in 25% (96) of study subjects. Down syndrome subjects were 2.4 times [aOR=2.416 (95%CI: 1.367–4.264)] more likely to die from sepsis. Those with associated comorbidities (Apert syndrome, Cerebral palsy, Chiari 2 malformation, Patau syndrome, Noonan syndrome, Congenital Rubella, Portal vein thrombosis, HIV, Scoliosis and VACTERL association) were 4.4 times more likely to die from sepsis [aOR=4.418 (95%CI: 1.617–12.072)].

**Conclusion:**

Bacterial sepsis is a common problem among children with CHD. Gram positive bacteria were common causes. Down syndrome and other co morbidities predicted bacterial sepsis mortality. Blood culture and sensitivity tests are recommended to halt the high mortality seen in Down syndrome or those with co morbidities.

## Introduction

Pediatric sepsis is a serious medical condition with estimated global incidence of 1.2 million cases. Its mortality is reported to range from 1 to 5% for sepsis and from 9 to 20% for severe sepsis (1). The Pediatric Sepsis Consensus Conference (PSCC) has defined sepsis taking into account the age specific vital signs and risk factors. Sepsis is defined as the presence of two or more systemic inflammatory response criteria, and confirmed or suspected invasive infection (2). Almost half of children with sepsis were reported to have underlying predisposing factor like chronic lung disease, congenital heart disease, malignancy or other neuromuscular disorders (3).

Blood culture positive rates among pediatric sepsis suspects varied between study settings with figures ranging from 22 to 35.7% (4–6). Gram-negative bacteremia was commonly documented. Staphylococcus aureus, pseudomonas, enterobacter species, Escherichia coli, and klebsiella were the common identified etiologic agents in pediatric sepsis (4–7). Children with congenital heart disease had higher incidence of culture positive sepsis, and staphylococcus was the common inciting organism (8).

Management of pediatric sepsis includes timey source control through surgical drainage or intervention, empiric antibiotic administration, fluid resuscitation, corticosteroid therapy and blood culture tests for etiologic agent and susceptibility profile assessment. Delayed treatment is associated with mortality (9).

Congenital heart disease (CHD) is a gross structural abnormality of the heart or the intrathoracic great vessels that is actually or potentially of functional significance (10). The current estimate for CHD incidence is 8–12/1000 live births. Countries with high fertility burden and low-quality health services are highly affected, and African countries are among the leads. While it is believed that timely correction of the CHD changes the sepsis risk, such CHD curative services are almost non-existent in most parts of Africa. The available services are supported by overseas mission-based volunteerism (11, 12). As to our review, there are no studies on sepsis among children with CHD in Africa, and from Ethiopia in particular. Our study aimed to assess the magnitude and determinants of bacterial sepsis among children with congenital heart disease.

## Methods

**Study area**: This study was conducted in Tikur Anbessa Specialized Teaching Hospital (TASH), Department of Pediatrics and Child Health, College of Health Sciences, Addis Ababa University, located in Addis Ababa, the capital city of Ethiopia. TASH is the largest and national medical referral center. The pediatric department provides outpatient and admitted care for a range of childhood illnesses. There are dedicated 115 pediatric beds (excluding neonatal intensive care unit) where admitted care is given for severe diagnosis with sepsis included. Children with CHD and suspected bacterial infection are admitted and investigated for possible etiologic agents. Investigations include blood culture, imaging like CXR and echocardiographic studies.

**Study design**: This was a retrospective cross-sectional study on the medical records of CHD patients, aged 2 months to 14 years, and admitted to TASH between 2017 and 2020.

**Study population**: All Children between the ages of 2 months and 14 years and who received admitted care between 2017 and 2020. A sample size of 384 was calculated using a single population proportion formula using the assumptions of 50% bacterial sepsis among CHD, 95% confidence interval and 5% margin of error. The proportion of bacterial sepsis of 50% was used as there were no previous studies in similar settings and to increase the power of the study by including the liberal maximum sample size. Consecutive cases fulfilling the inclusion criteria were included in the study until the final sample size was achieved.

Children with CHD and suspected sepsis, aged 2 months and 14years, and who received admitted care between 2017 and 2020 were included while those with incomplete medical records were excluded.

**Variables**: The dependent variable was patient outcome on hospital discharge (dead or alive). The independent variables include age, sex, type of CHD, other comorbidities, admission vital signs, presence of malnutrition, blood culture growth, ICU admission and duration of treatment.

**Data collection**: Structured questionnaire was prepared and data were collected from the medical records of patients and microbiology laboratory log book. Medical documents were retrieved from medical records archive.

**Operational definitions**: Anthropometric variables like height, weight, and weight for height were assessed and grouped as normal, stunted, underweight and wasted based on the WHO (World Health Organization) growth curves (13)] The vital signs were assessed based on the WHO, PSCC and PALS (pediatric advanced life support) charts, and grouped as normal, decreased and increased (2, 14, 15). The laboratory tests were grouped as normal, decreased and increased based on the age and gender references (16). Bacterial sepsis was considered when blood culture result revealed etiologic agent in the presence of clinical evidences (2).

**Data analysis**: Data entry and analysis were done on Statistical Software for Social Sciences (SPSS) v 24. Frequency and percentages were used to present categorical data. Binary logistic regression was done to assess the relation between dependent and independent variables. Variables with P value less than 0.25 on binary regression were taken into multiple nominal regression model for controlling the confounders while ascertaining possible associations. Finally, predictor variables were identified based on adjusted odds ratio (aOR), 95% confidence interval and P value less than 0.05.

**Ethical approval**: Ethical approval and waiver of consent for the study was obtained from the Pediatrics Research and Publication Committee, Department of Pediatrics and Child Health, Addis Ababa University.

## Results

**Sociodemographic characteristics**: The study included 384 children with slight female predominance, 54.2% (208). Most of study subjects were infants, aged 12 months and below, 59.1% (227). Half of the study subjects resided in the study area, 50.3% (193) while the rest, 49.7% (191), came from the other Ethiopian regional states (Table 1).

**Table 1:** Sociodemographic data of children admitted to TASH with CHD and sepsis, May 2017–July 2020

Variable	Frequency	Percent
***Age in months***		
2–6	124	32.3
7–12	103	26.8
13–59	129	33.5
60–144	28	7
***Gender***		
Male	176	45.8
Female	208	54.2
***Residence (by region)***		
Addis Ababa	193	50.3
Oromia	128	33.3
Amhara	29	7.6
SNNP&	23	6.0
Diredawa	5	1.3
Tigray	1	0.3
Benshangul Gumuz	2	0.5
Afar	1	0.3
Somali	2	0.5

& SNNP-Southern Nations and Nationalities Peoples regional state

**Clinical data**: Cough, fast breathing and fever were the common clinical presentations of sepsis among children with CHD, 66.9% (257). Patients with Down syndrome contributed to 26 % (100) study subjects. Acyanotic CHD was the common type of CHD, 66.7% (256). The most common type CHDs were ventricular septal defect (VSD) 25.5 % (98), atrial septal defect (ASD) 14.5% (56) and atrioventricular septal defect (AVSD) 13.5 % (52). Tachycardia, tachypnea and fever were documented in 90.1 % (346), 95.6(367) and 56.5 % (217) of study subjects, respectively. Oxygen saturation <95% was present in 90.1 % (346) of study subjects. Severe acute malnutrition was present in 26.3 % (101) of study subjects using weight for height (Table 2). None of our study subjects had undergone definitive treatment for their underlying cardiac illness.

**Table 2:** Clinical data of children admitted to TASH with CHD and sepsis, May 2017–July 2020

Variable	Frequency	Percentage
**Clinical presentation(n=384)**		
Cough, fast breathing and fever	257	66.9
Cough & fast breathing only	109	28.4
Other symptoms A	18	4.7
**History of previous hospital admission**(n=384)		
Yes	191	49.7
No	193	50.3
**Frequency of previous admissions**(n=191)		
≤2 times	176	92.1
> 2 times	15	7.9
**Type of CHD**(n=384)		
Cyanotic	128	33.3
Acyanotic	256	66.7
**Other Comorbidity**(n=384)		
Down syndrome	100	26.0
Other^B^	19	4.9
None	265	69
**Weight for age** (n=384)		
Normal	130	33.9
Between -2 & -3 Z score	82	21.4
< -3 Z score	172	44.8
**Height for age** (n=384)		
Normal	214	55.7
Between -2 & -3 Z score	37	9.6
< -3 Z score	133	34.6
**Head circumference for age** (n=384)		
Normal	224	58.3
<-3 Z score	48	12.5
Not documented	112	29.1
**MUAC** (n=384)		
Normal	101	26.3
Moderate acute malnutrition	22	5.7
Severe acute malnutrition	55	14.3
Not documented	206	53.6
**Weight/Height**(n=384)		
Normal	201	52.3
Between -2/-3 Z score	82	21.4
<-3 Z score	101	26.3
**WBC count for age**(n=384)		
Normal	297	77.3
Decreased	15	3.9
Increased	72	18.8
**RBC Count for age** (n=384)		
Normal	229	59.6
Decreased	33	8.6
increased	122	31.8
**Platelet count for age** (n=384)		
Normal	256	66.7
Increased	86	22.4
decreased	42	10.9

A Other symptoms include vomiting, diarrhea, failure to suck, loss of consciousness, easy fatigability and burning sensation during urination.

B Other comorbidities include Apert syndrome, Cerebral palsy, Chiari 2 malformation, Patau syndrome, Noonan syndrome, Congenital Rubella, Portal vein thrombosis, HIV, Scoliosis and VACTERL association

Eighty-nine percent (342) of study subjects were chest x-rayed. The most common chest x-ray finding was cardiomegaly 40.3% (155) followed by pneumonia 29% (112). Study subjects with documented abnormal chest x ray finding and oxygen saturation < 95% constituted 75.5% (290) of study population. There were only four cases of infective endocarditis out of which two had cyanotic heart disease.

**Figure 1:**
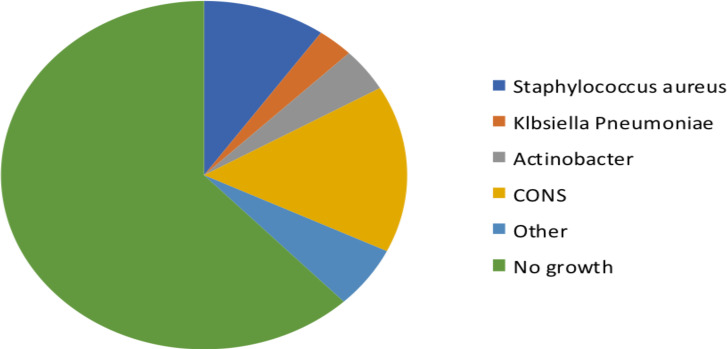
Blood culture isolates from children with CHD and sepsis at TASH, May 2017–July 2020 (**Other**: enterobacter, Escherichia coli, enterococcus fecalis, Bacillius, staphylococcus lugdunensis and species unidentified gram-negative rod & gram-positive cocci)

**Microbiology data**: A little more than a quarter blood culture sample, 28.1% (108) was taken within 24 hours of admission to the hospital. One third of subjects, 31.3% (120), had received antibiotics prior to the blood culture sample collection. Proportion of culture proven bacterial sepsis was 17.1 % (66) (95% CI: 13.6- 21.3). The most common isolated bacteria were coagulase negative staphylococcus aureus (CONS) 7% (27) followed by staphylococcus aureus 4.4 % (17) and actinobacteria 1.8% (7) (Figure 1). None of infective endocarditis cases had staphylococcus as an etiologic agent.

**Treatment and outcome**: Most of the study subjects were treated with different combination of drugs, 59.8% (230). Ceftriaxone was used in 35.4 % (136) of the study subjects (See table 3). Drugs switches for lack of adequate clinical response were the main reasons for prolonged duration of therapy in few subjects and mostly in ICU admitted cases. ICU admission was documented in 3.3 % (13) of the study subjects with median duration of stay of 5 days, and ranging from 1 to 85 days. Death occurred in 25 % (96) (95% CI: 20.7–29.6) of the study subjects and the remaining 75 % (288) were discharged alive.

**Table 3:** Treatment options used and length of treatment among CHD patients with sepsis at TASH: May 2017–July 2020

Variable	Frequency	Percent
**Treatment**		
Ceftriaxone	136	35.4
Vancomycin/cefepime	18	4.7
Other**	230	59.8
**Duration of treatment**		
<7 days	143	37.2
7–14days	197	51.3
15–21 days	16	4.2
>21 days	28	7.3

** Other Antibiotics include combination of ceftriaxone/gentamycin, ceftriaxone/vancomycin, Ampicillin/cefotaxime, Ampicillin/gentamycin, Meropenem and other drugs and with different combination like cloxacillin, crystalline penicillin, Metronidazole, ceftazidime, piperacillin tazobactam, ciprofloxacin, clindamycin.

**Predictors of treatment outcome**: Binary logistic regression was done to determine the relationship of treatment outcome with the independent variables. Multinomial logistic regression was used to control for the confounding factors and variables with P value < 0.25 on binary regression were included. Presence of Down syndrome and other comorbidities were associated with treatment outcome. Study subjects with Down syndrome were 2.4 times [aOR=2.416 (95% CI: 1.367–4.264)] more likely to die from bacterial sepsis when compared to those without Down syndrome. Study subjects with other comorbidities (Apert syndrome, Cerebral palsy, Chiari 2 malformation, Patau syndrome, Noonan syndrome, Congenital Rubella, Portal vein thrombosis, HIV, Scoliosis and VACTERL association) were 4.4 times more likely to die from sepsis than their counterparts [aOR=4.418 (95%CI: 1.617–12.072)]. Age, gender, type of CHD, presence of malnutrition, white blood cell count and type of treatment administered were not associated with bacterial sepsis treatment outcome (Table 4).

**Table 4:** Multiple logistic regression analysis: predictors of treatment outcome among CHD patients with sepsis at TASH, 2017–2020

Variable	Categories	Outcome		Adjusted Odds ratio with 95% CI	P value
		Discharge	Death		
Age in months	2–6	88	36	1.171(0.441–3.104)	0.752
	7–12	74	29	0.919(0.332–2.541)	0.870
	13–59	106	24	0.543(0.198–1.484)	0.234
	60–144	20	7	1	
Type of CHD	Cyanotic	103	25	0.881(0.488–1.591)	0.675
	Acyanotic	185	71	1	
Another comorbidity	Down syndrome	63	37	2.416(1.368–4.264)	0.002*
	Other$	9	10	4.418(1.617–12.072)	0.004*
	none	216	49	1	
MUAC	Normal	229	78	0.794(0.388–1.622)	0.526
	Moderate acute malnutrition	19	3	0.320(0.077–1.322)	0.115
	Severe acute malnutrition	40	15	1	
SaO2 (%)	<95	89	257	1.262(0.504–3.159)	0.619
	≥95	31	7	1	
Chest X ray findings	Normal	18	2	0.260(0.056–1.216)	0.087
	Pneumonia	88	24	0.623(0.319–1.217)	0.166
	Pulmonary edema	15	4	0.719(0.212–2.435)	0.596
	Cardiomegaly	112	43	0.912(0.496–1.676)	0.766
	Other&	55	23	1	

$ Apert syndrome, Cerebral palsy, Chiari 2 malformation, Patau syndrome, Noonan syndrome, Congenital Rubella, Portal vein thrombosis, HIV, Scoliosis and VACTERL association

& Boot shaped heart, right upper lobe collapse consolidation with hilar lymphadenopathy, cephalization with oligemic lungs, pleural effusion, TAPVC, left hemithorax homogenous opacity and cavitary lesion secondary to active TB

## Discussion

Bacterial sepsis among children with CHD is an understudied subject, particularly in Africa where CHD curative services are not readily available. Our study is the first to report the magnitude and determinants of bacterial sepsis treatment outcome among children with the nonintervened CHD from Ethiopia. Our study documented a higher bacterial sepsis occurrence among females and infants. Respiratory symptoms dominated the clinical picture while acyanotic CHD was the common type. One third of study subjects had received antibiotics prior to the blood culture sample collection. Severe acute malnutrition was documented in a quarter of study subjects. Culture proven bacterial sepsis was documented in 17% of study subjects with predominantly gram-positive etiology. Different combination of antimicrobials was used for treatment. A quarter of CHD children with sepsis had died. The presence of Down syndrome and other comorbidities predicted sepsis mortality among children with CHD.

In this study, infants have dominated the study population. This is in agreement with similar studies done in developing countries and western setting (8,17,18). This could be related to the early symptomatology of CHD in infancy as heart failure or sepsis, both requiring admission to a health facility.

Concerning gender, our study documented a higher bacterial sepsis occurrence among female children with CHD. Similar finding was reported from Egypt where females had higher hospital admission due to chest infections (19). This is as opposed to the documented evidence of a higher sepsis occurrence among boys owing to the hormonal and immunologic changes, and a more male CHD admission (17, 18, 20). While we couldn't forward the plausible reasons for this in our study, the above Egyptian study postulated a poor drug adherence among female CHD cases.

In our study, acyanotic CHD was the common type with ventricular septal defect being the commonest one. Similar pattern was documented in studies done in India, Nigeria, Egypt and Ethiopia (21–24). Children with more critical and cyanotic CHD could have died early due to the illness and or lack of access to the curative services. Moreover, the common complications seen among cyanotic CHD are hematologic abnormalities like polycythemia and anemia that can be managed as an outpatient case.

Respiratory symptoms like cough and fast breathing dominated the clinical presentation of sepsis in our study. The presence of underlying CHD was also documented as the strong predisposing factor for respiratory infection or pneumonia in children in other studies (25–27). Increased pulmonary blood flow and or compression of the airways are some of the mechanisms that lead to increased pulmonary infection or complication among children with CHD (27, 28).

In the current study, severe acute malnutrition was documented almost in a quarter of study subjects. This is lower than a previous Ethiopian study where almost half of children with CHD were reported to have SAM (29). Our study center is a national referral center where cases with nutritional deficiencies are seldom managed, and such cases are managed in the lower tier of Ethiopian health system ladder. Malnutrition among children with CHD is thought to be related to decreased intake and increased metabolism (30,31).

In the current study, culture proven bacterial sepsis was documented in almost one fifth of children with CHD and sepsis. There are no similar studies done in similar settings and with no CHD intervention for comparison. However, a western setting study documented sepsis in about 6% of children with CHD. But this study only included young infants up to the age of 4 months. Generally, comparable culture positivity rates for bacterial sepsis suspects were reported from developing countries (4,5). Concerning the etiologic agents, gram positive bacteria were the common isolated agents in our study. Similar pattern was documented in the above western study (8).

In the current study, the case fatality rate of bacterial sepsis was 25%. There are no studies done in related settings to compare this figure. However, a western study documented a mortality rate of 4 %. The difference could be noted for the difference in institutional capacity and inclusion of post-operative subjects in the later study (8).

This study documented presence of Down syndrome and other comorbidity as strong predictors of bacterial sepsis treatment outcome among children with CHD. Study subjects with Down syndrome had doubled odds of death from sepsis when compared to their counterparts. A similar finding was reported in another study (32). This could be due to immunologic impairments associated with Down syndrome i.e., chemotactic defects, decreased IgG4 levels, and quantitative and qualitative abnormalities of the T cell and B cell systems. The healthcare provider and parents' clinical treatment decision difference between children with and without Down syndrome could also be postulated (32, 33). The presence of other comorbidities like Apert syndrome, Cerebral palsy, Chiari 2 malformation, Patau syndrome, Noonan syndrome, Congenital Rubella, Portal vein thrombosis, HIV, Scoliosis and VACTERL association increased the odds of sepsis related death by fourfold. Such comorbidities were the common documented associated findings among children with CHD (34). These comorbidities affect the immune system through genetic syndromes, structural malformations, metabolic associations and teratogens, and increase the risk of sepsis and associated death among children with CHD (35).

The limitation of this study includes its retrospective nature with limitations to assess all factors associated with bacterial sepsis. Moreover, there could be synergistic effect of co morbidities and or syndromes and CHD on sepsis burden. Our study could not assess this due to the retrospective nature of the study. However, this study reviewed and assessed predictors of bacterial sepsis among CHD in resource limited setting and as to our search it's the first of its kind in Africa and Ethiopia. Moreover, this study included large sample size. In terms of informing the health policy, our study output could serve as a resource data for planning CHD interventions and to guide activities to limit sepsis among children with CHD.

In conclusion, bacterial sepsis is a common problem among children with congenital heart disease. Acyanotic CHD with female predominance and respiratory symptoms was the common presentation pattern. Almost one-fifth culture proven bacterial sepsis was documented. Gram positive bacteria were the commonly isolated etiologic agents ending in a quarter of deaths. Presence of Down syndrome and other associated comorbidities predicted the outcome of bacterial sepsis among children with CHD. CHD children with Down syndrome and other comorbidities should be thoroughly evaluated for bacterial sepsis and investigated including blood culture, and treatment should be geared by antimicrobial susceptibility profile.
